# RNA interference-mediated knockdown of voltage-gated sodium channel (*MpNa*_*v*_) gene causes mortality in peach-potato aphid, *Myzus persicae*

**DOI:** 10.1038/s41598-019-41832-8

**Published:** 2019-03-28

**Authors:** Kaleem Tariq, Asad Ali, T. G. Emyr Davies, Erum Naz, Laila Naz, Summar Sohail, Maolin Hou, Farman Ullah

**Affiliations:** 1 Department of Agriculture, Abdul Wail Khan University, Mardan, Khyber Pakhtunkhwa, Pakistan; 2 Department of Biointeractions and Crop Protection, Rothamsted Research, Harpenden, Hertfordshire, AL5 2JQ UK; 3 State Key Laboratory of Agricultural Microbiology; Hubei Key Laboratory of Insect Resource Application and Sustainable Pest Control, Huazhong Agricultural University, Wuhan, 430070 Hubei China; 4 State Key Laboratory for Biology of Plant Diseases and Insect Pests, Institute of Plant Protection, Chinese Academy of Agricultural Sciences, Beijing, 100193 China; 5 Department of Entomology, College of Plant protection, China Agriculture University, Beijing, 100193 China

## Abstract

Voltage-gated sodium channels (VGSC) are transmembrane proteins that generate an action potential in excitable cells and play an essential role in neuronal signaling. Since VGSCs play a crucial role in nerve transmission they have become primary targets for a broad range of commercial insecticides. RNA interference (RNAi) is a valuable reverse genetics tool used in functional genomics, but recently, it has also shown promise as a novel agent that could be used to control agricultural insect pests. In this study, we targeted the VGSC (*MpNa*_*v*_) gene in the peach-potato aphid *Myzus persicae*, by oral feeding of artificial diets mixed with dsRNAs. Knock-down of *MpNa*_*v*_ gene expression caused up to 65% mortality in 3^rd^ instar nymphs. Moreover, significantly lower fecundity and longevity was observed in adult aphids that had been fed with dsMpNa_v_ solution at the nymphal stage. Analysis of gene expression by qRT-PCR indicated that the aphid mortality rates and the lowered fecundity and longevity were attributable to the down-regulation of *MpNa*_*v*_ by RNAi. Taken together, our results show that *MpNa*_*v*_ is a viable candidate target gene for the development of an RNAi-based bio-aphicide.

## Introduction

The peach-potato aphid *Myzus persicae* (Hemiptera: Aphididae) is a major sap-sucking insect pest that infest more than 400 species of plants belonging to 40 different families including Brassicaceae and Solanaceae. Owing to direct plant feeding and the transmission of more than 100 plant viruses, it is considered one of the most destructive agricultural pests worldwide^1,2^. Its broad host range, telescoping of generations and cyclical parthenogenesis make it a highly successful insect pest^3^. Synthetic chemical insecticides are considered the most effective way to combat *M*. *persicae*. However, it has developed resistance against several different insecticide classes^4^, resulting in significant control failures and losses in protected crops.

An appropriate functioning of the voltage-gated sodium channel (VGSC) is essential for the normal transmission of the nerve impulse in insects, including aphids, and disruption of the action potential by insecticides leads to paralysis and eventually death of the insect^5^. The insecticides that target the VGSC have broad spectrum effects, as the structure of the VGSC is highly conserved across the animal kingdom, so there is a consequential detrimental impact on non-target species including pollinators and other beneficial’s. There is thus a real need to develop alternative (species specific) control methods with lower environmental impacts^6^.

RNAi is a genetically conserved post-transcriptional gene silencing mechanism that facilitates down regulation of gene expression by small noncoding RNA molecules in almost all eukaryotes^7,8^. RNAi has over the past decade been developed as a molecular tool to knock-down target gene transcripts in insects^9^. Due to its high degree of specificity, RNAi is also being widely explored as a potential novel pest control strategy for a variety of pest species^10^, with the perceived benefits of increased species discrimination and decreased risk to the environment and non-target species^11^.

Unusually in *M*. *persicae* (and other aphids), the functioning VGSC is encoded by two genes (NCBI Accessions FN601405 and FN601406)^12^ with some unique properties that are not present in the VGSCs of other insects. Unlike the channels of other insects, the aphid has a unique heterodimeric channel (composed of two subunits, H1 and H2, encoding DI-II and DIII-IV of the VGSC respectively) with an atypical ion selectivity filter (similar that found in the mammalian sodium sensor channel Na_x_)^13^, which, atypically for insect VGSCs, is extremely insensitive to tetrodotoxin.

Abd El Halim *et al*.^14^ recently reported that RNAi-mediated knock-down of VGSC gene expression, through application of complementary dsRNA, caused significantly high larval mortalities and severe developmental arrest in the red flour beetle *Tribolium castaneum*, a coleopteran insect pest that has proved to be particularly amenable to RNAi^15,16^. In comparison levels of gene-knock down and systemic RNAi responses (following injection or ingestion of dsRNA) in many other insect classes are extremely variable^17^. In particular, RNAi outcomes in phloem sap feeding hemipteran species such as aphids, whitefly, psyllids and plant hoppers can be exceedingly disparate, ranging from no phenotype to significant mortality and from very low to complete gene knock-down^18–20^. Hemipteran species are therefore more challenging to work with as they appear to be somewhat intractable to RNAi manipulations^21^. In this study, we demonstrate that oral delivery of *MpNa*_*v*_ dsRNA to the peach-potato aphid *M*. *persicae* successfully down-regulates the expression of the aphids VGSC and causes significant nymphal mortalities. Moreover, lower fecundity and longevity was also observed in dsRNA treated insects. These results suggest that RNAi targeting the VGSC could be a promising novel bio-pesticide against this hemipteran pest.

## Materials and Methods

### Insect culture

*Myzus persicae* were collected from a cabbage field in Mardan, Khyber Pakhtunkhwa, Pakistan, and established as a laboratory colony that was maintained on cabbage plants (variety Golden Acre) under standard laboratory conditions (25 ± 2 °C, 70 ± 10% relative humidity, 12:12 (light: dark) photoperiod).

### Total RNA extraction and cDNA synthesis

Total RNAs were isolated from 1^st^, 2^nd^, 3^rd^, 4^th^ instar nymphs and adults of *M*. *persicae* using Wizol™ Reagent (Wizbiosolutions Inc., Korea) following the manufacturer’s recommended protocol. RNA integrity was analyzed on 1.5% (w/v) agarose gels as described in Sambrook and Russell^22^. cDNA’s were synthesized using 1 μg total RNA with WizScript™ First Strand cDNA synthesis kit (Wizbiosolutions Inc. Korea), according to the manufacturer’s recommended protocol.

### Synthesis of dsRNA molecules

For RNAi experiments, a 289 bp fragment of the *M*. *persicae MpNa*_*v*_ (heteromer H1) gene (NCBI Accession FN601405) and a 329 bp fragment of the *Aequorea victoria* green-fluorescent protein (GFP) gene (NCBI Accession M62653) were amplified from cDNA obtained from adult aphids using PCR. The T7 promoter sequence 5′GGATCCTAATACGACTCACTATAGGA3′ was added in front of the forward and reverse PCR primers as required for subsequent dsRNA synthesis (Table 1). Primers were chosen based on the results of GeneScripts primer design software (https://www.genscript.com/tools/pcr-primers-designer). The reaction mixture for PCR contained 500 ng of the cDNA template, 0.5 µm of forward and reverse primers, 1.75 mM Mg^2+^, 0.25 mM of each dNTP (Takara Bio, Japan), 1X PCR buffer, 5U/µl Taq DNA polymerase (Takara Bio) and double-distilled H_2_O to make a 25 µl total reaction volume. The PCR conditions were 95 °C for 5 min, followed by 35 cycles of 95 °C for 30 s, 55–60 °C for 30 s and 72 °C for 30 s, and an additional final polymerization step of 72 °C for 5 min. PCR products were purified using TIANgel Midi Purification Kit (Tiangen, Beijing, China). These purified products were then used to synthesize dsRNA using the T7 RiboMAX™ Express RNAi System (Promega, US), following the manufacturer’s protocol. The dsRNA quality was monitored on agarose gel electrophoresis, and the concentration was determined by spectrophotometry (Nano Drop 1000, Thermo Scientific, US). dsRNA products were stored at −80 °C prior to further use.

**Table 1:** Primers used for dsRNA synthesis and for qRT-PCR.

Primers	Primer sequence
**dsRNA synthesis**	
dsMpNa_v_ –F	**GGATCCTAATACGACTCACTATAGGA** CGACGACTCGAACGCTGTGA
dsMpNa_v_ -R	**GGATCCTAATACGACTCACTATAGGA** CCCGCACTCGTCCACTTGTT
dsGFP-F	**GGATCCTAATACGACTCACTATAGGA** AGAGTGCCATGCCCGAAGGT
dsGFP-R	**GGATCCTAATACGACTCACTATAGGA** AAGGACAGGGCCATCGCCAA
**qRT-PCR**	
MpNa_v_ –F	AAGCAATCCGAGCGAAACTC
MpNa_v_ –R	CCATCCCGTCACCAATTGTC
Actin-F	GGTGTCTCACACACAGTGCC
Actin-R	CGGCGGTGGTGGTGAAGCTG

### Dietary delivery of the double-stranded RNA to nymphs

The artificial feeding diet for *M*. *persicae* was prepared as described by Pan *et al*.^23^, albeit with a 20% lower sucrose content^24^. In the RNAi diet, dsMpNa_v_ or dsGFP RNA, or DEPC water was mixed in and fed to 3^rd^ instar nymphs. To rear aphids on this artificial diet, 6 well culture plates were used with small ventilation holes incorporated in the bottom of the plates. Using a fine paintbrush, one hundred and fifty 3^rd^ instar nymphs were carefully collected from cabbage leaves and transferred to each well of the plate, and the plate sealed with Parafilm^®^M. 0.75 mg/ml^25^ dsMpNa_v,_ or dsGFP RNA, or DEPC water was incorporated into the artificial diet as required, and the mixture loaded on the parafilm membrane above the wells; feeding sachets were created with 2 cm^2^ pieces of parafilm, cleaned with ethanol and DEPC water. Employing this strategy, the aphids could puncture the inner layer of parafilm membrane and feed on the mixture of diet sandwiched between the two layers of parafilm. The plates were kept under laboratory conditions (25 ± 2 °C, 70 ± 10% RH, and 12:12 (light: dark) photoperiod) and the diet mixtures renewed every day for 7 days. Mortality of the aphids was recorded daily. All bioassay treatments were repeated in triplicate.

### *MpNa*_*v*_ expression analysis by Quantitative Real-Time PCR

Quantitative reverse transcription PCRs (RT-qPCR) was performed to analyze the expression level of *MpNa*_*v*_ (heteromer H1). Primers for the *MpNa*_*v*_ H1, *GFP* and *Actin* genes were designed online using Primer-BLAST^26^ (https://www.ncbi.nlm.nih.gov/tools/primer-blast/). qRT-PCR reactions were performed using iTaq™ Universal SYBR Green Supermix (Bio-Rad) in a Bio-Rad iCycler (Bio-Rad, Hercules, CA, USA), following the manufacturer’s instructions. PCR conditions were 95 °C for 10 min, 40 cycles of 95 °C for 15 s, 60 °C for 30 s and 72 °C for 30 s. All the analyses were repeated in triplicate. Relative expression levels were calculated using 2−△△Ct method^27^. *Actin* was used as the internal control^28^. All the primers used in this study (Table 1) were designed to avoid the homologous ORF region used for the synthesis of dsRNA.

### Longevity and fecundity analysis

After continuous feeding of dsRNA for 7 days, each survivor aphid was transferred to a cabbage leaf placed on a freshly prepared agar plate. Plates were kept under laboratory condition as described above. The number of new born aphid nymphs from each plate was recorded daily until the aphid died. The nymphs produced by the aphids were removed from the dishes after counting. The cabbage leaf was replaced every alternate day with a fresh leaf.

### Statistical analysis

The data were statistically analyzed with GraphPad prism 5.0. The results were analyzed using One-way analysis of variance (ANOVA) with subsequent Tukey Kramer multiple comparison. For all tests, *P* < 0.05 was considered significant.

## Results

### Temporal expression of *MpNa*_*v*_ gene

**Figure 1:**
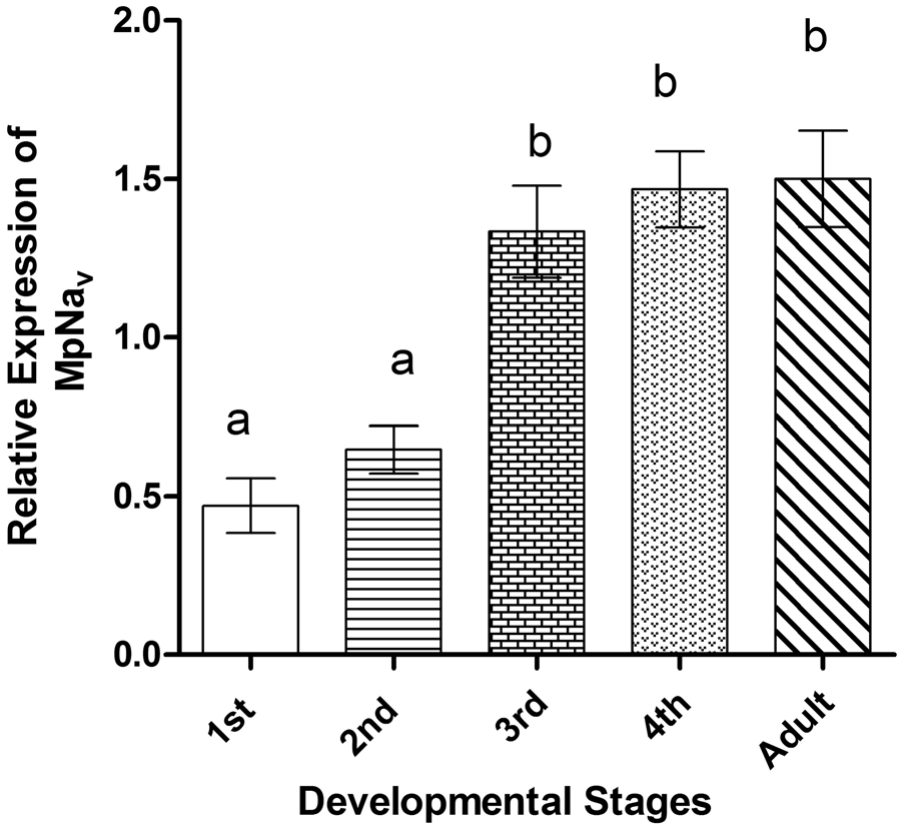
Stage-specific expression pattern of the *M*. *persicae* gene *MpNa*_*v*_ H1 in the whole insect body analyzed by qRT-PCR. The letters above the bars show significant differences (least significant difference in one-way analysis of variance, P < 0.05) in *MpNa*_*v*_ expression. Means with the same letter are not significantly different. Mean ± SEM of three independent experiments are shown. RNA was normalized with Actin as the internal control.

The relative abundance of *MpNa*_*v*_ H1 mRNA at specific developmental stages of *M*. *persicae* was estimated by qRT-PCR. Transcripts were detected in all life stages investigated. However, MpNa_v_ H1 was most abundantly expressed (*P* < 0.005) in 3^rd^ and 4^th^ instar nymphs and adults (Fig. 1). There was no significant difference in the level of *MpNa*_*v*_ gene expression in the 3^rd^ instar, 4^th^ instar, and adult aphids. Based on this, 3^rd^ instar nymphs were selected as a suitable developmental stage for RNAi studies.

### Bioinformatic analysis of targeted sequence

Homology of the selected MpNa_v_ dsRNA fragment to other insect Na_v_ sequences, including representatives from important pollinator and beneficial species, was investigated using the NCBI BLASTn algorithm. The BLASTn search indicated a high degree of similarity between the MpNa_v_ dsRNA fragment and the Na_v_ coding sequence in other aphid species (overall homology scores ranging between 95% identity, e-value 5e-125 to the pea aphid *Acyrthosiphon pisum* to 84% identity, e-value 3e-83 to the sugar-cane aphid *Sipha flava*). For important pollinating and beneficial insects, Blastn scores ranged from 74% identity, e-value 2e-16 identity for the common eastern bumble bee, *Bombus impatiens*, 73% identity, e-value 1e-13 for the common honey bee, *Apis mellifera*, and 71% identity, e-value 1e-12 for the parasitic wasp *Trichogramma pretiosum*.

### *MpNa*_*v*_ expression after dsRNA feeding

**Figure 2:**
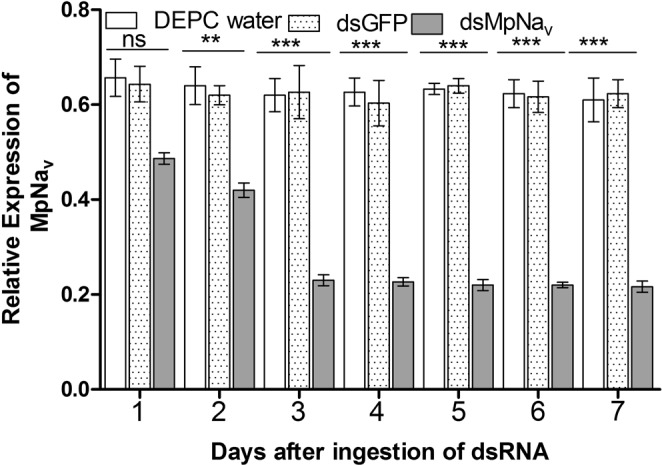
Dietary delivery of *MpNa*_*v*_ dsRNA alters *MpNa*_*v*_ expression in *M*. *persicae*. Relative abundances of *MpNa*_*v*_ H1 gene transcripts, as determined by qRT-PCR of cDNA made from total RNA isolated from whole body 1 to 7 days after the indicated dietary treatments of *M*. *persicae*. Data represent the mean ± SEM of three independent experiments. *MpNa*_*v*_ mRNA was normalized with *Actin* as an endogenous control. Treatment was compared with controls using ANOVA (Tukey Kramer multiple comparison, p < 0.05). Symbols ns, ** and *** indicates non-significant, P < 0.01 and P < 0.005 respectively.

The silencing efficiency of dsMpNa_v_ was examined using real-time quantitative PCR. We analyzed expression of *MpNa*_*v*_ at daily intervals following continuous oral delivery of dsRNA. A direct correlation was observed between the amounts of dsRNA ingested and a consequent decrease in the abundance of MpNa_v_ mRNA transcript. At the 2nd day of ingestion, MpNa_v_ dsRNA caused significant (*P* < 0.01) reduction in *MpNa*_*v*_ mRNA abundance. During the 3^rd^ to 7^th^ day of dsMpNa_v_ feeding the *MpNa*_*v*_ transcript level showed very significant differences (*P* < 0.001, 2.5-fold decrease) compared with the DEPC water and dsGFP control treatments, indicating a substantial gene knockdown (Fig. 2).

### Effects of dsRNA on aphid development and mortality

**Figure 3:**
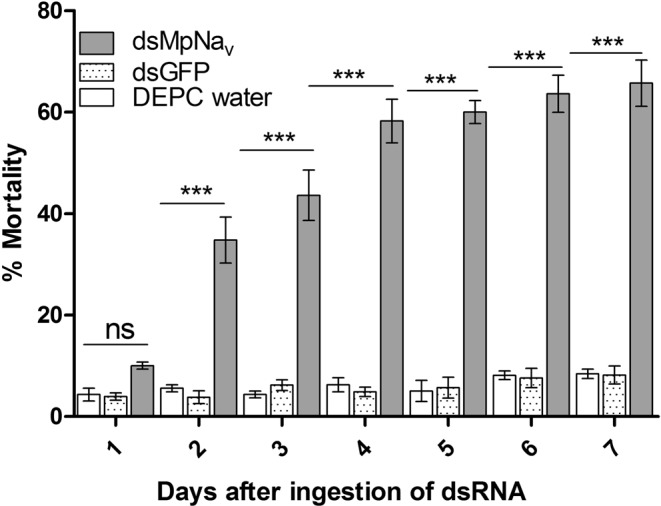
Mortality rate of *M*. *persicae* fed artificial diets. Mortality rates by feeding artificial diets containing DEPC water, dsGFP and dsMpNa_v_ over time. Mean ± SEM of three replications (n = 150) are shown. Different treatments were compared using ANOVA (Tukey Kramer multiple comparison, p < 0.05). Symbols ns and *** indicates non-significant and P < 0.005 respectively.

**Figure 4:**
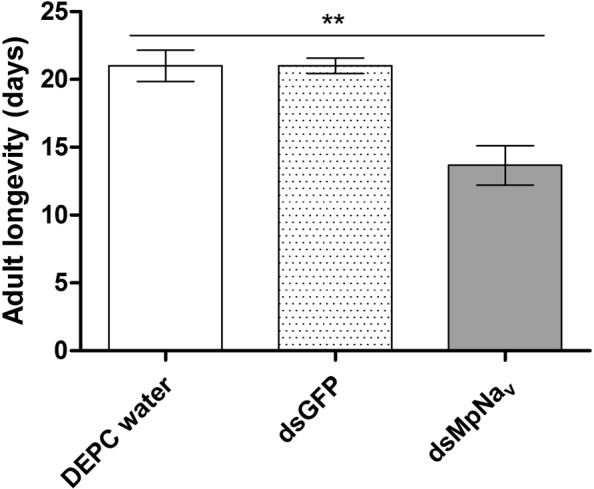
Effect of *MpNa*_*v*_ silencing on adult longevity of *M*. *persicae*. Life-spans of insects fed on artificial diets containing DEPC water, dsGFP and dsMpNa_v_ at their nymphal stage. Mean ± SEM of three replications (n = 20, 18, or 17) are shown. Treatments are compared using ANOVA (Tukey Kramer multiple comparison, p < 0.05). Symbol ** indicates P < 0.01.

**Figure 5:**
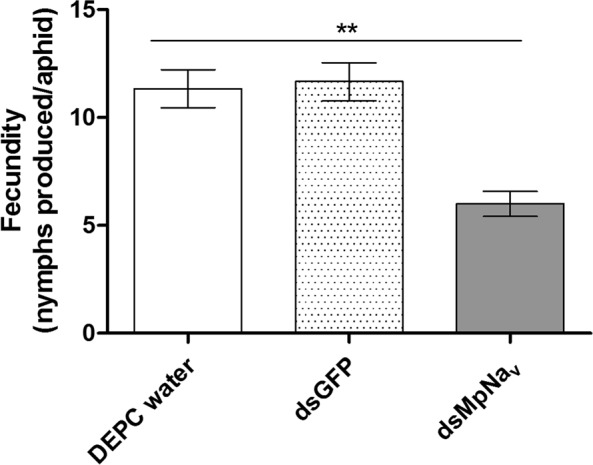
Effect of *MpNa*_*v*_ knock-down on fecundity of *M*. *persicae*. Fecundity of insects previously fed on artificial diets containing DEPC water, dsGFP and dsMpNa_v_ at their nymphal stage. Mean ± SEM of three replications (n = 18) are shown. Treatments are compared using ANOVA (Tukey Kramer multiple comparions, p < 0.05). Symbol ** indicates P < 0.01.

No significant aphid mortality was observed for the DEPC water, dsGFP and dsMpNa_v_ treatments after the first day of feeding. The mortality differences between treatment become more and more obvious from the 2^nd^ day of feeding onwards. The average mortality for dsMpNa_v_ treatments reached 34.7%, 43.6%, 58.2%, 60.0%, 63.6% and 65.7% respectively after the second, third, fourth, fifth, sixth and seventh day of continuous feeding, whereas the mortality of the DEPC water treated group was 5.5%, 4.3%, 6.2%, 5.0%, 8.1% and 8.4% respectively, whilst the mortality for dsGFP oral feeding treatments was 3.8%, 6.2%, 4.8%, 5.7%, 7.6% and 8.1% respectively (Fig. 3). Relative to the -ve (DEPC water) and +ve (dsGFP) control diets, which exhibited only a small decrease in survival over an assay period of 7 days, the dsMpNa_v_ containing diet resulted in a significant (p < 0.005) impact on aphid mortality between days 3 and 7. At day 7, any surviving insects were transferred to cabbage leaves and their longevity and fecundity evaluated. Prior dietary delivery of dsMpNa_v_ significantly decreased (p < 0.01, p < 0.05) the longevity and fecundity of *M*. *persicae* survivors compared to control treatments (Figs 4 and 5).

## Discussion

For electrical signaling in eukaryotes, VGSCs are essential and ubiquitous^29^. Insect VGSCs are normally encoded by a single gene (*para* in *Drosophila melanogaster* and its equivalent orthologs in other insect species), that can generate a large number of transcriptional editing and splice variants that are differentially expressed at various developmental stages within the life cycle and in specific cell types^30^. Unusually in *M*. *persicae* (and other aphids), the functioning VGSC is encoded by two genes (designated heteromer H1 and H2) with some unique channel properties^13^. These two heteromer’s are hypothesized to have arisen due to a gene inversion event having occurred at some time during the evolution of aphids, resulting in a novel two-subunit channel.

Abd El Halim *et al*.^14^ recently demonstrated that RNAi-mediated knockdown of a VGSC gene (*TcNa*_*v*_) by oral feeding causes up to 51.34% larval mortality and developmental arrest in the red flour beetle *T*. *castaneum*. This dose dependent larval mortality was observed after the beetles were continuously fed dsRNA for 6 days. Moreover, adult emergence was also significantly reduced in insects fed with dsRNA. Our results for the peach-potato aphid channel *MpNa*_*v*_ are on a par with this study, with up to 63.6% mortality observed for aphid nymphs by day 6 of exposure to dsRNA targeted against the H1 subunit. In our experiments, knock-down of the *MpNa*_*v*_ gene also significantly reduced adult longevity (by up to 7 days) and the fecundity (a decline of approximately 45%) of survivors. A previous study in *Drosophila melanogaster* has reported that decreased levels of the *para* VGSC in mle^napts^ (no-action potential temperature sensitive mutation of the maleless (mle) gene) mutant flies resulted in decreased longevity and fecundity^31^. mle^napts^ is a recessive gain-of-function mutation of mle that results in a splicing defect of the Na^+^ channel transcript and a severe reduction of VGSC RNA levels and channel activity. Another recent RNAi study targeting the VGSC in the bird cherry-oat aphid *Rhopalosiphum padi*^32^, a global pest of wheat, indicated significant suppression of the transcript levels of heteromers H1 and H2, in this case by direct injection (rather than oral feeding) of their respective dsRNA, and a significant cross-suppressions in the transcript levels between H1 and H2 subunit genes. Although in our investigations we did not analyse expression of the *M*. *persicae* H2 subunit in response to application of H1 dsRNA, the likelihood is that significant cross-suppression will also have occurred.

If we compare the regions targeted for RNAi suppression in these three individual studies, whereas in *T*. *castaneum* a 239 nt dsRNA covering the membrane spanning regions S1 and S2 in Domain I of the channel (GenBank accession NM_001165908.1) was used, the two separate aphid studies (*M*. *persicae* and *R*, *padi*) employed dsRNAs encompassing the DI-DII linker region of heteromer H1. For *R*. *padi* H1 (GenBank accession KJ872633) a larger 485 bp *Rpvgsc1* fragment was amplified. However, in both aphid studies a common region of 71 amino acids (214 nucleotides) was covered in the amplified fragments. In both cases similar levels of suppression were reported, suggesting that this region is a good target for RNAi suppression technology. In the *R*. *padi* study a 358 bp dsRNA fragment encompassing the DIV S4-S5 and S5-S6 linker region (*Rpvgsc2* GenBank accession no. KP966088) of heteromer H2 (which is the more evolutionarily divergent gene when compared to classical monomeric VGSCs) was also selected for suppression studies. It is not, however, clear from these experiments which region of the gene (coding region 3′ or 5′ end) is ideal for dsRNA design, and in fact it may not really be that important e.g. in the pea aphid *Acyrthosiphon pisum*, no difference in mortality was observed in groups of insects fed with dsRNA matching either the 5′ or 3′ end of the hunchback (hb) gene^25^.

Unintentional gene silencing in non-target species^33^ is the primary risk posed by pesticidal RNAi. Bioinformatic analyses that compares the pesticidal RNAs to non-target genomes has been recognised as an useful initial screen that can help to predict potential non-target risks posed by RNAi^33,34^. In the present study, we used *in-silico* searches to determine whether our 289 bp dsRNA shared prohibitive sequence similarities with the genes from key insect pollinators and beneficial insects. We obtained somewhat similar homology scores in the range 73–74% for the bee species represented in the NCBI nucleotide database and the highest score for a beneficial insect (predatory wasp) was 71%. In comparison, other aphid species returned high homology scores of 84–95%, suggesting that in practice the dsRNA used in this study may suppress the VGSC expression of more than one aphid type. No significant hits were obtained when the off-target search algorithm dsCheck^35^ was used to identify exact and near nucleotide matches of the 289 bp *M*. *persicae* dsRNA to the genome of the model insect *D*. *melanogaster*. However, even considerable sequence divergence between an mRNA and the dsRNA does not rule-out unintentional gene silencing since, once ingested, the dsRNA is cleaved into numerous very short (19–23 nucleotides) small interfering RNAs (siRNA). These siRNAs most likely have abundant direct sequence matches throughout most eukaryotic and prokaryotic genomes, thereby increasing the chances for off-target (i.e. silencing of a gene with sufficient sequence similarity to the dsRNA) and non-targeted (silencing of the intended gene in an unintended organism) effects^36^.

E-RNAi^37^ analysis used for design of the dsRNA used in the *T*. *castaneum* study^14^ predicted over two hundred 19 nt siRNAs could be generated from the 239 bp dsRNA fragment (albeit with an average efficiency score of 54.16, and no off-target predictions); there is thus considerable scope for unforeseen off-target effects. Most studies report that dsRNA ranging from 140 to 500 nucleotides in length are required for successful RNAi in insects, although successful suppression with dsRNA of 50 bp has been reported^38^. This finding could be helpful to further optimize the dsRNA specificity by using shorter amplified dsRNA fragments, thereby decreasing the likelihood of non-target and off-target effects. The success of RNAi is also dependent on the molecules stability and an effective uptake of the dsRNA by the target species^39^. A rapid degradation of dsRNA by extracellular ribonucleases in the insect haemolymph and gut is increasingly recognised as a fundamental factor influencing RNAi efficiency in several insect orders^40^. This is exacerbated in hemipteran species since extra-oral salivary degradation of dsRNAs provides an additional block to cellular uptake^17,21,41^. To translate RNAi to field applicability, RNAi silencing elicitors will most likely need to be combined with biotic or abiotic systems that mediate both protection and uptake of the eliciting RNAi trigger^39^.

To conclude, our study demonstrates that silencing of a voltage-gated sodium channel gene (*MpNa*_*v*_) via RNAi in *M*. *persicae* caused larval mortality. It provides evidence to propose that *MpNa*_*v*_ is a feasible candidate gene to target for the control of this insect pest using RNA interference technology and provides the foundation to design dsRNA molecules and associated delivery systems with a high degree of species specificity that could ultimately be used as commercial bio-aphicides.

## Data Availability

All data generated or analysed during this study are included in this published article.
